# Exposure to oestrogen and risk of anastomotic leakage after colorectal cancer surgery – A clue to the different leak rates in men and women

**DOI:** 10.1111/codi.16300

**Published:** 2022-09-13

**Authors:** Martin Rutegård, John Moshtaghi‐Svensson, Caroline E. Weibull, Ulrika Ottander, Caroline Nordenvall, Malin Sund

**Affiliations:** ^1^ Department of Surgical and Perioperative Sciences, Surgery Umeå University Umeå Sweden; ^2^ Wallenberg Centre for Molecular Medicine Umeå University Umeå Sweden; ^3^ Clinical Epidemiology Division, Department of Medicine Solna Karolinska Institutet Stockholm Sweden; ^4^ Department of Clinical Sciences, Obstetrics and Gynecology Umeå University Umeå Sweden; ^5^ Department of Molecular Medicine and Surgery Karolinska Institutet Stockholm Sweden; ^6^ Department of Pelvic Cancer, GI Oncology and Colorectal Surgery Unit Karolinska University Hospital Stockholm Sweden; ^7^ Department of Surgery University of Helsinki and Helsinki University Hospital Helsinki Finland

**Keywords:** anastomotic insufficiency, gender, hormones, sex

## Abstract

**Background:**

Colorectal anastomotic leakage is consistently more common in men, regardless of tumour location. This fact is largely unexplained but might be a consequence of biological differences including hormonal exposure and not only related to anatomy.

**Methods:**

This was a retrospective, nationwide registry‐based observational study of post‐menopausal women operated for colorectal cancer with an anastomosis between 2007 and 2016. Hormonal exposure before surgery, as defined by prescribed drugs affecting oestrogen levels, was related to postoperative anastomotic leakage, using mixed‐effects logistic regression models with adjustment for confounding. Odds ratios (ORs) with corresponding 95% confidence intervals (CIs) were derived. In addition, separate estimates according to tumour location were computed, and a sensitivity analysis excluding topical oestrogen hormone exposure was conducted.

**Results:**

Some 16,535 post‐menopausal women were included, of which 16.2% were exposed to drugs increasing oestrogen levels before surgery. In this exposed group compared to the unexposed, leak rates were 3.1 and 3.8%, respectively. After adjustment, a reduction of anastomotic leakage in the exposed group was detected (OR: 0.77; 95% CI: 0.59–0.99). This finding was largely attributed to the rectal cancer subgroup (OR: 0.55; 95% CI: 0.36–0.85), while the exclusion of topical oestrogen drugs further reduced the estimates of the main analysis (OR: 0.63; 95% CI: 0.38–1.02).

**Conclusions:**

Anastomotic leakage rates are lower in women exposed to hormone replacement therapy before surgery for colorectal cancer, which might explain some of the difference in leak rates between men and women, especially regarding rectal cancer.

**What does this add to the literature?**

Women experience less anastomotic leakage after colorectal cancer surgery than men, a hitherto unexplained fact. This observational study investigates different levels of preoperative oestrogen hormone exposure, suggesting that oestrogen increasing drugs might reduce the incidence of anastomotic leakage.

## INTRODUCTION

Colorectal anastomotic leakage is a major complication, often resulting in morbidity and sometimes mortality [1, 2]. The aetiology of leakage is largely unexplained, though considered multifactorial in nature. Numerous risk factors have been evaluated, where a distal anastomotic location and male sex consistently display a strong association to leakage [1, 3]. A meta‐analysis estimated a 50% higher anastomotic leakage incidence in men [3]. In rectal cancer specifically, the lower leak rates in women have been explained by a technically easier dissection, due to a wider pelvis. Recent pelvimetric studies have however shown that, despite an association between larger pelvic inlet and outlet and leakage, male sex remains an independent risk factor for leakage [4]. Moreover, this sex‐specific effect also exists in colon cancer surgery, possibly due to dissimilarities in visceral fat distribution [5]. Hormonal differences between men and women are considered important in other biological processes, where antiandrogens and oestrogens have been shown to positively affect skin wound healing [6]. While such research is sparse for colorectal anastomotic healing, experimental data suggest that male mice deposit less collagen in colonic anastomoses [7]. To evaluate the suggestion that female sex hormones play a role in leakage aetiology, a population‐based study was conducted. In this study, it was hypothesized that exposure to oestrogen in women might decrease the risk of colorectal anastomotic leakage, in comparison to nonexposed women.

## METHODS

### Checklist for the reporting of observational studies

This article was written in accordance with the Strengthening the Reporting of Observational Studies in Epidemiology (STROBE) checklist for the reporting of observational studies [8].

### Data source

A nationwide registry study was conducted based on *Colorectal Cancer Base Sweden (CRCBaSe)*, a mega‐linkage originating from a national quality register, *the Swedish Colorectal Cancer Registry (SCRCR)*, that contains all Swedish patients diagnosed with colorectal cancer. For rectal cancer, the registration started in 1995 and since 2007 the registry also incorporates patients with colon cancer. The national compliance in 2008–2015 was 98.5 and 98.8% for colon and rectal tumours, respectively [9]. Data are prospectively recorded during treatment and follow‐up. Patient and tumour characteristics such as age, sex, American Society of Anesthesiologists' grade, tumour location and tumour stage are reported in detail, as well as preoperative treatment and perioperative data including type of surgery and postoperative complications. Several other registries are incorporated into CRCBase and described here. *The Prescribed Drug Registry* reports information on all prescribed and dispensed drugs since July 2005 [10]. Medications sold over‐counter or given in‐hospital cannot be identified in this registry. The registry contains information on Anatomical Therapeutic Chemical (ATC) code, dosage, and date of prescription as well as withdrawals. *The National Patient Registry* includes dates of diagnosis and surgery and codes for different diagnoses. Data on inpatient care has been collected since 1964 (with nationwide coverage since 1987) and outpatient data since 2001, with a national coverage of more than 99% [11]. Information regarding educational attainment was retrieved from *the Longitudinal Integrated database for Health Insurance and Labour Market Studies (LISA) database* [12]. *The Registry of Total Population* was used for information on migration [13] and *the Cause of Death Registry* for information about vital status.

### Study design

This is a retrospective observational study, where women diagnosed with colorectal cancer in 2007–2016 were identified in the SCRCR. The inclusion criteria comprised colorectal cancer surgery with an anastomosis and age over 50 years, as the latter indicated menopause [14]. Clinical data, including tumour data, surgical and oncological treatment, were derived from the SCRCR, while comorbidity and education data were collected from the National Patient Registry and the LISA database, respectively. Mortality was derived from the Cause of Death Registry.

### Study exposure and outcome

#### Exposure

The exposure was classified according to ATC codes derived from the Prescribed Drugs Registry. To be classified into either group of oestrogen decrease or increase, the relevant drugs had had to be withdrawn at least two times consecutively, of which at least one time within 6 months before the colorectal cancer operation. Oestrogen increase drugs comprised agents from the following ATC groups: G03CA03, G03CA04, G03CC07, G03CX01, G03FA, and G03FB. These drugs constituted hormonal replacement therapy, including topical vaginal therapy. Oestrogen decrease comprised tamoxifen, fulvestrant, and aromatase inhibitors, with the following ATC codes: L02BA01, L02BA03, L02BG03, L02BG04, and L02BG06. Patients simultaneously fulfilling criteria for the oestrogen increase group and the oestrogen decreasing drug were excluded from the former but included in the latter group. Unexposed women did not fulfil criteria for neither the oestrogen increase nor the oestrogen decrease group.

#### Outcome

Anastomotic leakage within 30 days or in‐hospital was the primary outcome measure. There is no formal definition of leakage in the registry, but this variable has been validated concerning rectal cancer and found to be underreported (29%) when compared to an international consensus definition [15], while virtually no false positives were found [16]. Of note, there is no information available on how and when anastomotic leakage was diagnosed within the above time frame, whereas any reintervention for leakage is recorded (ranging from procedures under local anaesthesia, for example, percutaneous drainage, to relaparotomy/relaparoscopy).

#### Tumour location

Right‐sided cancers included any cancer from the caecum to the transverse colon; left‐sided cancers included the splenic flexure down to the sigmoid; and rectal cancers were defined as any cancer with the inferior border within at least 15 cm from the anal verge, as measured by a rigid sigmoidoscope.

### Statistical analysis

**FIGURE 1:**
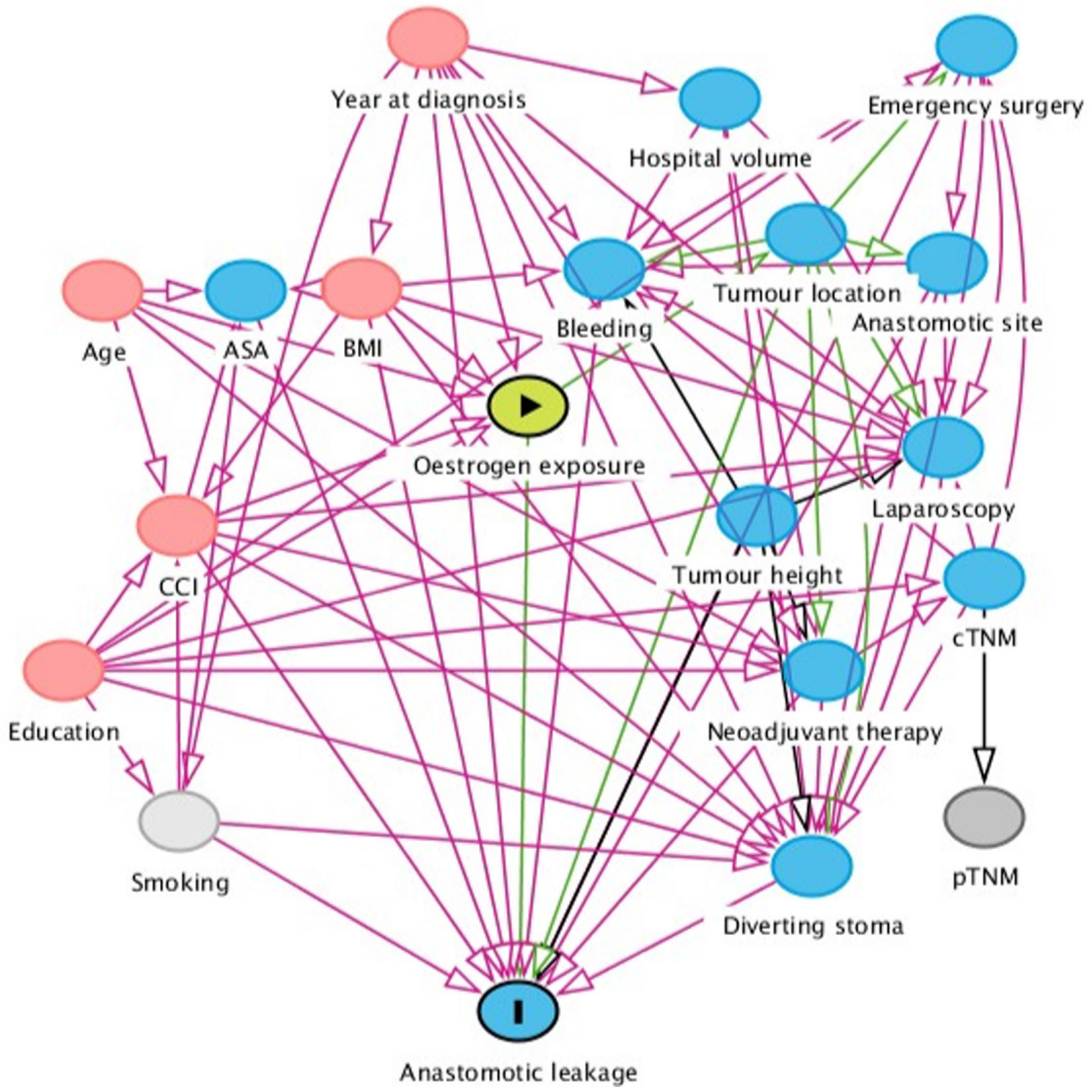
Directed acyclic graph, depicting the proposed relationships between exposure, outcome and other variables pertaining to the research question. Red circles indicate ancestors of both outcome and exposure, necessary to adjust for to eliminate confounding. ASA, American Society of Anesthesiologists' physical fitness grade; BMI, body mass index; CCI, Charlson comorbidity index; c/pTNM, clinical/pathological tumour‐node‐metastasis staging system

A directed acyclic graph [17] was used to help select potential confounding factors for the multivariable analyses, aiming to estimate the total effect of oestrogen exposure on the risk of anastomotic leakage (Figure 1). These factors comprised a minimally sufficient adjustment set, whereas adjustment for more covariates, for example, perioperative variables, would bias the estimates and decrease precision, as these are not simultaneously unidirectionally related to both exposure and outcome. The selected covariates were age at diagnosis, calendar year of surgery, body mass index (BMI), Charlson Comorbidity Index (CCI) [18], and educational attainment; the first three variables were included as continuous variables in the models, while BMI and formal education were treated as categorical variables (<25, 25–30, or >30 kg/m^2^; and <9, 9–12, or >12 years, respectively). Unadjusted logistic regression as well as mixed‐effects logistic regression analyses with adjustment for the above covariates, also including operating hospital as random effect, were performed, producing odds ratios (ORs) with 95% confidence intervals (CIs). Also, an interaction model was used to estimate separate effects of oestrogen exposure according to different tumour locations. In addition, a sensitivity analysis was conducted to evaluate the impact of excluding women with topical hormone replacement therapy only. A complete cases analysis was used throughout, as missing data was rare. The statistical analyses were performed in R version 4.1.1.

#### Power

Prior to data collection, a power calculation was derived. The risk of anastomotic leakage in women operated for colorectal cancer was assumed to be 5%, while the use of hormone replacement therapy in menopause has previously been described at 10% in Swedish women [19]. With a further assumption that the oestrogen increase group would have a reduced proportion of leakage by half, 7880 patients were required to prove such a difference at a 5% significance level with 90% power.

## RESULTS

### Study participants

**FIGURE 2:**
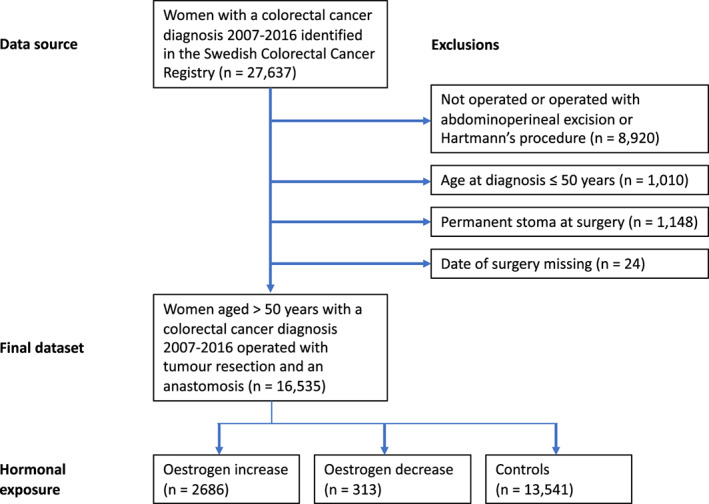
Flowchart of study inclusion and exclusion with a depiction of hormonal exposure groups

In this study, 16,535 women were included. Of these, 2686 (16.2%) had prescription withdrawals consistent with oestrogen increase, 313 (1.9%) with oestrogen decrease, while 13,541 (81.9%) women were considered unexposed (Table 1, Figure 2). Of note, most clinical and demographic variables were similarly distributed between exposure groups, though comorbidity (due to a prior breast cancer diagnosis) was more prevalent in the oestrogen decrease group. Moreover, a substantial majority of the oestrogen increase group was ascribed to this group due to topical hormone replacement only (70%).

**TABLE 1:** Demographic and clinical data for 16,535 women aged over 50 years and operated for colorectal cancer during 2007–2016 in Sweden, divided by different levels of oestrogen exposure from prescription drugs

Variables	Women controls (*N* = 13,541)	Oestrogen increase (*N* = 2686)	Oestrogen decrease (*N* = 313)
Categorical	*N* (%)	*N* (%)	*N* (%)
Body mass index (kg/m^2^)			
<25	6233 (46%)	1285 (48%)	140 (45%)
25–30	3777 (28%)	768 (29%)	96 (31%)
>30	1955 (14%)	353 (13%)	50 (16%)
Formal education (years)			
<9	4241 (31%)	720 (27%)	88 (28%)
9–12	6322 (47%)	1240 (46%)	145 (46%)
>12	2788 (21%)	700 (26%)	79 (25%)
Tumour location			
Right colon	7822 (58%)	1527 (57%)	188 (60%)
Left colon	3668 (27%)	771 (29%)	77 (25%)
Rectum	2045 (15%)	385 (14%)	48 (15%)
Missing	6 (0%)	3 (0%)	0 (0%)
Pathological tumour stage			
0‐I	2344 (17%)	575 (21%)	56 (18%)
II	4802 (35%)	912 (34%)	109 (35%)
III	4503 (33%)	891 (33%)	98 (31%)
IV	1764 (13%)	310 (12%)	48 (15%)
Missing	128 (1%)	28 (1%)	2 (1%)
Emergency surgery	1906 (14%)	310 (12%)	40 (13%)
Minimally invasive surgery	1966 (15%)	448 (17%)	49 (16%)
Mortality within 90 days	517 (4%)	67 (2%)	12 (4%)

Continuous	Median (IQR)	Median (IQR)	Median (IQR)
Age (years)	74 (66–81)	73 (66–80)	74 (66–81)
Charlson Comorbidity Index	0.0 (0.0–1.0)	0.0 (0.0–2.0)	2.0 (2.0–6.0)
Year of diagnosis	2011 (2009–2014)	2012 (2009–2014)	2012 (2009–2014)

*Note*: Percentages may not add up due to rounding.

Abbreviation: IQR, interquartile range.

### Anastomotic leakage by oestrogen treatment

For unexposed women, the leak proportion was 3.8%, while the oestrogen increase and decrease groups had shares of 3.1 and 2.6%, respectively (Table 2). Of these, reintervention for leakage amounted to 72, 82, and 75%, respectively. The adjusted analyses showed a significant result only for the oestrogen increase group, with an OR of 0.77 (95% CI: 0.59–0.99). In analyses stratified by tumour location, this effect was detected only in the rectal cancer group (OR: 0.55 [95% CI: 0.36–0.85]). In the sensitivity analysis excluding those using topical hormonal replacement only, a more pronounced risk reduction, though not statistically significant, was found compared with the main analysis (OR: 0.63 [95% CI: 0.38–1.02]); in this analysis, the oestrogen increase and decrease groups had leak proportions of 2.8 and 2.6%, respectively.

**TABLE 2:** Anastomotic leakage as a function of oestrogen exposure in women aged over 50 years and operated for colorectal cancer, as well as stratified by tumour location

Oestrogen group	Leakage proportion	Unadjusted OR (95% CI)	Adjusted^a^ OR (95% CI)
All locations			
No exposure	518/13,541 (3.83%)	Reference	Reference
Oestrogen increase	83/2,684 (3.09%)	0.80 (0.63–1.01)	0.77 (0.59–0.99)
Oestrogen decrease	8/310 (2.58%)	0.66 (0.30–1.25)	0.75 (0.37–1.55)
Right colon			
No exposure	227/7822 (2.90%)	Reference	Reference
Oestrogen increase	38/1527 (2.49%)	0.85 (0.59–1.19)	0.78 (0.50–1.20)
Oestrogen decrease	3/188 (1.60%)	0.54 (0.13–1.44)	0.60 (0.18–1.96)
Left colon			
No exposure	146/3668 (3.98%)	Reference	Reference
Oestrogen increase	30/771 (3.89%)	0.98 (0.24–2.66)	0.97 (0.63–1.50)
Oestrogen decrease	3/77 (3.90%)	0.98 (0.64–1.44)	1.09 (0.33–3.57)
Rectum			
No exposure	145/2045 (7.09%)	Reference	Reference
Oestrogen increase	15/385 (3.90%)	0.53 (0.30–0.88)	0.55 (0.36–0.85)
Oestrogen decrease	2/48 (4.17%)	0.57 (0.09–1.87)	0.57 (0.17–1.87)

*Note*: Mixed‐effects logistic regression was used to derive odds ratios (ORs) with 95% confidence intervals (CIs).

^a^ With adjustment for age at diagnosis, Charlson Comorbidity Index, body mass index, educational attainment, and year of diagnosis.

## DISCUSSION

In this nationwide study of female post‐menopausal colorectal cancer patients, a potential risk reduction in anastomotic leakage was detected as a function of recently prescribed drugs increasing oestrogen levels. This decrease was more pronounced in the group with oral oestrogen treatment. The effect was mainly evident in rectal cancer patients, and might provide a clue to the observed differences in anastomotic leakage between men and women.

There are limitations to the present study. Misclassification of both exposure and outcome is probable. The exposure classification assumes that withdrawals equal drug intake, which is impossible to control. By excluding those with only one prescription withdrawal the chance of patients actually taking the drug was increased. Information of oestrogen‐containing creams and herbal remedies was not available. There is also a known underreporting of leakage in the SCRCR; however, this nondifferential misclassification probably leads to attenuation of associations rather than the opposite. There was no information about whether the anastomosis was hand‐sewn or stapled. Although clinically interesting, it is difficult to conceive how the anastomotic technique would affect the exposure and can therefore hardly be considered a confounder. However, there is a risk of residual confounding, as in all observational studies.

Strengths include the population‐based design, which alleviates selection bias. In addition, high‐quality data on patient and tumour characteristics were available, including potential confounders such as BMI and comorbidity. The nationwide capture also provides an ample sample size, necessary to evaluate even small anticipated outcome differences.

While the sex difference in anastomotic leakage has long been known, the literature is surprisingly devoid of studies evaluating possible causes. Disparities in rectal cancer surgery are not explained by differing pelvic dimensions [4], and leak rates are higher in men even in colon cancer operations [3]. While experimental data point to biological differences regarding e.g. collagen deposition [7], and there are indications that visceral fat contributes to differing leak frequencies [5], the present study is the first attempt to evaluate the effects of oestrogen‐modulating drugs on the risk of anastomotic leakage in women. Contrary to the study hypothesis, the oestrogen decrease group did not have higher rates of leakage; however, the number of women in this group was low, prohibiting any firm conclusions. This finding could also be related to differences in cellular responses of oestrogen receptor (ER) blockage as the major intestinal receptor is ERβ. In experimental settings, this receptor is associated with inflammation, hypoxia and epithelial permeability in the colon. Moreover, oestrogen receptor modulators have been shown to exert both antagonistic and agonistic effects on ERβ, whereas oestrogen itself appears to have mostly agonistic affects [20, 21]. Another mechanism may be the possible association between gut microbiome and anastomotic leakage [22]. Hormonal changes alter the microbiome in women [23], and experimental studies have shown a positive correlation between intestinal ERβ and microbiome diversity [24].

Further research is warranted, as colorectal anastomotic leakage is a major clinical problem, and the role of oestrogen modulation could be important. The study results might potentially be used to better stratify women to anastomotic surgery, or to avoid an unnecessary defunctioning stoma in rectal cancer surgery.

## AUTHOR CONTRIBUTIONS

Martin Rutegård and Malin Sund conceived the study idea, while Martin Rutegård, Ulrika Ottander, Caroline Nordenvall and Malin Sund developed the study design. Martin Rutegård, John Moshtaghi‐Svensson, Caroline E. Weibull and Caroline Nordenvall acquired the data. The data was then prepared by Caroline E. Weibull and John Moshtaghi‐Svensson. Statistical analysis was conducted by John Moshtaghi‐Svensson, while data interpretation was made by Martin Rutegård, Caroline E. Weibull, Caroline Nordenvall and Malin Sund. Martin Rutegård drafted the manuscript, while John Moshtaghi‐Svensson, Caroline E. Weibull, Ulrika Ottander, Caroline Nordenvall and Malin Sund edited and critically revised the manuscript. All authors gave final approval of the published version, and agreed to be accountable for this work.

## FUNDING INFORMATION

Knut and Alice Wallenberg Foundation, Swedish Society of Medicine, Cancer Research Foundation in Northern Sweden, the Stockholm Cancer Society, and the Regional Agreement on Medical Training and Clinical Research between the Stockholm County Council and Karolinska Institutet.

## CONFLICT OF INTEREST

None declared.

## ETHICAL APPROVAL

The study was approved by the Regional Board of the Ethical Committee in Stockholm, Sweden (DNR: 2014/71–31, 2018/328–32, 2021–00342).

## Data Availability

The data that support the findings of this study are available from the Swedish Colorectal Cancer Registry. Restrictions apply to the availability of these data, which were used under license for this study. Data are available from the author(s) with the permission of the Swedish Colorectal Cancer Registry.
